# The adaptive ecological trap: a grounded theory study of adolescent AI dependency

**DOI:** 10.3389/fpsyg.2026.1839672

**Published:** 2026-06-18

**Authors:** Gaochang Gong, Guoqiang Li

**Affiliations:** School of Education, Hunan University of Humanities, Science and Technology, Loudi, Hunan, China

**Keywords:** adaptive ecological trap, adolescents, artificial intelligence, grounded theory, technology dependency

## Abstract

**Introduction:**

This study explores the formation mechanisms of adolescent dependency on artificial intelligence and proposes a theoretical model of the “adaptive ecological trap” to explain its dynamic evolutionary process.

**Methods:**

Employing a grounded theory approach, in-depth interviews were conducted with 38 secondary school students who frequently used generative AI, and the data were analyzed through three-level coding.

**Results:**

The results indicated that the formation of adolescent AI dependency is a systemic process that originates from gaps in real-world developmental needs and initiates compensatory use under the combined influence of technological allure and individual-environmental vulnerabilities. According to participants' accounts, this process may be perceived to erode key developmental capacities and potentially contribute to a self-reinforcing cycle. The model delineates a four-stage evolutionary pathway from adaptive use to increasingly impairing dependency: trap laying, triggering, tightening, and locking. This study emphasizes that AI dependency is not merely a behavioral addiction but an outcome of dysregulation within the individual-technology-environment ecosystem, characterized by dynamism, interactivity, and self-reinforcing properties.

**Discussion:**

The findings call for collaborative interventions involving families, schools, technology platforms, and social policies. Furthermore, measures such as filling real-world developmental gaps, enhancing individual and environmental resilience, and disrupting negative feedback loops are crucial to assist adolescents in achieving healthy, autonomous development in the digital era.

## 1 Introduction

Artificial intelligence (AI), particularly generative AI, is integrating into adolescents' daily lives with unprecedented breadth and depth (Xiaokang, 2025; Hongyan, 2025). Beneath the halo of technological empowerment, AI casts a shadow, fostering dependency and eroding user agency. The American Psychological Association (American Psychological Association, 2025) issued a health advisory, noting that adolescents may form unhealthy emotional attachments owing to excessive immersion in parasocial relationships with AI. The phenomenon of using AI as a primary problem-solving tool and regarding it as a core emotional support is increasingly common, raising widespread concerns among scholars concerning the potential impairment of adolescents' critical thinking, social-emotional development, and long-term autonomy.

In this study, “AI dependency” refers to a pattern of behavior characterized by prioritized and frequent use of generative AI, subjective difficulty in moderating use, and self-perceived functional interference in academic, social, or emotional domains (operationalized in the Methods section). This construct differs from internet addiction, smartphone addiction, and social media addiction (Young, 1998; Griffiths and Kuss, 2017), which typically involve generalized digital media use without the specific relational and cognitive affordances of generative AI. It also differs from emotional attachment to chatbots (American Psychological Association, 2025), which may occur without functional impairment. Unlike problematic technology use broadly defined, AI dependency emphasizes the compensatory and self-reinforcing loop between unmet real-world needs and technological allure, as detailed in our proposed model. This distinction is critical for avoiding conceptual overlap and for designing targeted interventions.

Previous studies revealed AI's potential risks from multiple perspectives. At the micro level, studies focused on the “cognitive outsourcing” enabled by AI, which may weaken metacognition and deep thinking abilities (Ortega-Ochoa et al., 2024; Machidon, 2025; Pergantis et al., 2025). At the macro level, discussions addressed the long-term shaping of adolescents' social mindsets by algorithmic culture (Xiaokang, 2025). However, extant studies had two key limitations. First, explanations remained static, failing to clearly describe how various factors dynamically intertwine and progressively evolve into a complete dependency formation process. Second, theories were fragmented, lacking a core model that organically integrates the “individual-technology-environment” triad to reveal the inherent cyclical logic through which dependent behaviors self-perpetuate and deteriorate. In sociology, a “trap” refers to a systemic dilemma in which actions taken by actors to cope with immediate pressures are rationally sound in the short term but lead to a worsening of their developmental situation, from which escape is difficult (Xuming et al., 2020). Drawing on this metaphor, this study proposed the concept of an “adaptive ecological trap” to characterize a specific dilemma that begins as adaptive use but progressively alienates into problematic dependency within systemic interactions. Consequently, this study aimed to examine whether adolescents' dependency on AI constitutes such an adaptive ecological trap. The term “ecological” is adopted from Bronfenbrenner's ecological systems theory (Bronfenbrenner, 1979), which emphasizes that human development is shaped by nested environmental systems (micro-, meso-, exo-, and macro-systems). In our model, adolescent AI dependency is not an individual pathology but an outcome of dysregulation within the individual-technology-environment ecosystem, where reciprocal interactions among personal vulnerabilities, AI affordances, and inadequate support systems create a self-reinforcing trap.

Notably, adolescence is a critical period for identity formation, impulse control, and the development of emotional regulation and social skills (Griffiths and Kuss, 2017). Neurodevelopmental changes render adolescents particularly sensitive to reward cues and immediate gratification—features that AI systems are designed to exploit. Moreover, adolescents face mounting academic pressures and a need for autonomy, which may increase their vulnerability to compensatory technology use. These developmental characteristics make adolescents a priority group for investigating AI dependency.

## 2 Literature review

### 2.1 Evolution of theoretical perspectives: from behavioral addiction to compensatory use

The research trajectory on technology dependency evolved from an initial medical framework of behavioral addiction, focusing on clinical features, such as uncontrolled use and withdrawal symptoms, to theories emphasizing psychosocial compensation (Young, 1998). Owing to the advent of AI possessing highly realistic social capabilities, the explanatory power of purely behaviorist approaches has limitations. The Compensatory Internet Use Model, proposed by Kardefelt-Winther (2014), marked a significant theoretical shift, suggesting that excessive online behavior may not stem from technology's addictive nature but rather from attempts to compensate for unmet psychosocial needs in life. Considering generative AI's powerful appeal among adolescents through this lens, its roots lie in the technology's ability to provide both cognitive and emotional compensation. Regarding cognitive function compensation, Ortega-Ochoa et al. (2024) identified 962 records in their initial search, but after screening, only four studies were included in their mini-review. Within these, they found that generative AI tools may enhance student classroom participation and learning interest, although the limited number of included studies precludes strong conclusions about statistical significance. Pergantis et al. (2025) systematically reviewed AI's potential and current state in the cognitive domain, suggesting positive prospects for enhancing cognitive flexibility, attention, working memory, problem-solving, and metacognition. However, concerns exist that long-term reliance on generative AI may weaken the brain's capacity for deep information processing and long-term memory consolidation, impairing metacognitive abilities (Deckker and Sumanasekara, 2025). However, a more profound impact might be hindering the development of adolescents' independent problem-solving and analytical reasoning skills, leading to a phenomenon termed “intellectual deskilling” (Machidon, 2025).

Regarding emotional function compensation, generative AI serves as a low-risk, highly controllable “ideal partner”. Through algorithmic analysis of user input, it provides seemingly precise, empathetic, and tirelessly responsive feedback. This undoubtedly offers a safe, non-judgmental emotional outlet for adolescents experiencing social anxiety or loneliness (Xie et al., 2025). Previous studies indicated that a significant portion of users consider AI chatbots as “intimate others” for seeking comfort and companionship, with much interaction involving sharing worries and emotional catharsis (Le and Huimin, 2026). However, this algorithm-based “parasocial relationship” has a fundamental limitation: it may substitute for, rather than supplement, human relationships. Long-term reliance on such patterned, pre-programmed emotional responses might diminish individuals' capacity to perceive, understand, and respond to complex emotions in social settings, particularly causing a degradation of empathetic ability.

Building on the compensatory framework, Zhai et al. (2025) proposed a dual-path model in which instrumental motivations (e.g., information seeking, efficiency) predict cognitive reliance, while affective motivations (e.g., companionship, loneliness) predict emotional attachment—two distinct pathways to chatbot dependency. Moreover, Kim et al. (2025) found that relational conversational AI (personified, empathetic) appeals more to adolescents than transparent AI, particularly among those with lower quality real-world relationships. These findings align with our emphasis on the interplay between technological affordances (B) and individual-environmental vulnerabilities (C).

Recent years have seen the development of validated instruments that allow researchers to differentiate between non-problematic frequent use, instrumental reliance, emotional attachment, and functional impairment. The AI Addiction Scale (AIAS-21) assesses problematic AI use along seven behavioral addiction criteria (Pantic, 2026). The AI Dependence Scale (AIDep-22) distinguishes four dimensions—emotional dependence, functional dependence, cognitive dependence, and loss of control—with good reliability among Chinese undergraduates (Wu et al., 2026). The Generative AI Awareness Scale for secondary school students captures knowledge, positive attitudes, and concerns about AI (Semerci Sahin et al., 2025). These tools support the view that AI engagement is heterogeneous and that dependency exists on a continuum rather than as a binary condition.

### 2.2 An integrative process analysis framework: the “ecological trap”

The concept of the “trap” provides an integrative framework for understanding such systemic dependency phenomena. In social sciences, a “trap” is characterized as an adaptive action taken by an individual to cope with immediate difficulties, which, in the long run, leads them into a worse and inescapable situation (Xuming et al., 2020). Introducing this concept into technology-dependency research offers three theoretical values. First, it emphasizes the process's dynamic and paradoxical nature, focusing on how dependency evolves from reasonable adaptive use. Second, it emphasizes the system's interactive characteristics, emphasizing that trap formation results from the complex interplay of individual traits, technological affordances, and environmental support systems. Third, it implies the outcome's locking-in nature: the erosion of key developmental capacities caused by initial dependency further exacerbates the original real-world difficulties, forming a cycle of “gap → compensation → erosion → larger gap,” ultimately locking in the dependent state. Packin and Chagal-Feferkorn (2024) aligned with the trap theory framework, suggesting that AI's addictive design, when combined with individual and environmental vulnerabilities (e.g., family, school), can produce synergistic amplification of risks. However, extant studies have yet to systematically integrate these disparate threads into a dynamic, interactive, cyclical “ecological trap” analytical picture. Therefore, this study aimed to construct an integrative theoretical model, grounded in the empirical data of Chinese adolescents, to fill this gap.

It is also important to acknowledge that AI use is not inherently harmful. A recent meta-analysis found that AI chatbots can effectively reduce depression and anxiety in adolescents and young adults (standardized mean difference −0.35 for depression and anxiety) (Feng et al., 2025). Thus, the adaptive ecological trap model does not pathologize AI use but rather specifies the conditions under which adaptive use may turn maladaptive. The present study acknowledges both the potential benefits and the risks, aiming to inform a nuanced understanding.

## 3 Research design and data analysis

### 3.1 Research methodology

This study strictly adhered to the classic grounded theory methodology established by Glaser and Strauss (1967) and systematized by Strauss and Corbin (1990). This methodology eschews presupposed theoretical frameworks, advocating for an open-minded approach that allows theory to “emerge” from empirical materials. This bottom-up construction process emphasizes the gradual formation of a theoretical model explaining the phenomenon through constant comparison, conceptualization, and abstraction (Strauss and Corbin, 1990).

### 3.2 Data collection

Data were collected between June 2025 and October 2025 through semi-structured interviews with 38 adolescents (Table 1) using purposive and theoretical sampling. Initial purposive sampling presupposed four criteria: (1) currently enrolled secondary school student (grades 7–12); (2) frequent use of generative AI for more than 6 months; (3) exhibiting a tendency toward dependency; and (4) providing informed consent.

**Table 1:** Interviewees' basic information distribution (*N* = 38).

Variable	Category	Number	Percentage
Gender	Male	20	52.6
	Female	18	47.4
Grade	7th	6	15.8
	8th	8	21.1
	9th	5	13.2
	10th	7	18.4
	11th	7	18.4
	12th	5	13.2
Family location	Urban	21	55.3
	Town/Rural	17	44.7

### 3.3 Operational definition and identification of “AI dependency tendency”

In this study, “AI dependency tendency” was conceptually defined as a pattern of adolescent–AI interaction characterized by three core features: (a) prioritized and frequent use—the adolescent regularly turns to generative AI as a first-line solution for cognitive or emotional needs, often bypassing alternative human or self-driven strategies; (b) subjective difficulty in moderating use—a perceived loss of control or emotional discomfort (e.g., irritability, restlessness) when access to AI is limited; and (c) functional interference—self-perceived or observable negative consequences in academic performance, real-world social relationships, or emotional well-being that are attributed to AI use. This conceptualization deliberately distinguishes dependency from mere high-frequency use, instrumental convenience, or situational reliance (Kardefelt-Winther, 2014; Young, 1998).

Identification of participants who exhibited a “tendency toward dependency” followed a two-stage, multi-informant process:

**Stage 1—Teacher nomination (initial screening)**. Homeroom teachers from participating middle schools were provided with a one-page behavioral anchor guide (see Supplementary Material S1) listing observable signs consistent with the above conceptualization. Examples included: “the student frequently uses generative AI to complete assignments that peers finish independently without AI”; “the student shows noticeable anxiety, frustration, or protest when advised to limit AI use”; “the student prefers conversing with AI chatbots over face-to-face social activities after school”. Teachers were instructed to nominate students who had exhibited at least two of these indicators during the preceding two months. This step served to identify a pool of potentially relevant cases and was not intended as a clinical diagnosis.

**Stage 2—Researcher**-**administered structured screening (confirmation)**. Each nominated student then completed a brief, orally administered screening checklist (see Supplementary Material S2) developed for this study. The checklist comprised six simple yes/no items derived directly from the three core features of dependency:

1. When facing a difficult problem (study or personal), do you usually go to AI first before trying to solve it yourself?
2. Do you use generative AI almost every day, and feel uncomfortable if you miss one day?
3. Do you feel upset, restless, or bored if you cannot use generative AI for a day?
4. Has your use of AI ever caused your grades to drop, or made you spend less time with friends/family?
5. Do you find it difficult to stop using AI even when you know you have other important things to do?
6. Have you ever lied to your parents or teachers about how much you use AI?

Students who answered “yes” to at least four of the six items were considered to exhibit a clear dependency tendency and were invited to participate in the full in-depth interview. All screenings were conducted individually by the second author (Guoqiang Li), a trained researcher in developmental psychology, in a private room at the participant's school. The term “tendency” was retained intentionally to acknowledge a continuum of severity and to avoid premature pathologizing. This two-stage process was applied uniformly to all 38 participants. The six additional participants recruited during theoretical sampling underwent the same confirmation procedure before being included.

### 3.4 Recruitment procedure

Participants were recruited from two public secondary schools in central China (one urban, one peri-urban). The research team first obtained approval from the school principals and the district education bureau. Nominated students received a written information sheet explaining the study purpose, procedures, confidentiality, and voluntary nature. For students under 18 (all participants), parental written consent was obtained before the student's own assent was sought. Recruitment continued until theoretical saturation was reached, as described in the “Theoretical sampling and piloting” subsection below.

### 3.5 Interview procedures and materials

All interviews were conducted in person by the first author (Gaochang Gong), a trained researcher in developmental psychology, in a quiet, private room at the participant's school (e.g., an unused classroom or a counseling office). No teachers or parents were present during the interviews to minimize social desirability bias. Each interview lasted approximately 55 min on average (range: 40–75 min) and followed a semi-structured interview guide (see Supplementary Material S3 for the full guide). The guide covered four main domains: (1) AI usage history and patterns (e.g., “Which AI tools do you use? For what purposes? How often?”); (2) motivations and perceived benefits (e.g., “Why do you turn to AI rather than other sources of help?”); (3) emotional and cognitive experiences with AI (e.g., “How do you feel when interacting with AI? Do you feel it understands you?”); and (4) self-perceived negative consequences and reflections (e.g., “Has AI use ever caused problems for you? Have you tried to cut down?”). The guide allowed flexible probing to explore emergent themes.

Language, recording, and transcription: All interviews were conducted in Mandarin Chinese (the native language of both the interviewer and the participants). Sessions were audio-recorded using a digital recorder with the participants' verbal consent. The recordings were transcribed verbatim by a professional transcription service, and the transcripts were then checked for accuracy by the second author. To prepare English-language quotations for this manuscript, the relevant excerpts were translated from Chinese to English by the first author (Gaochang Gong), a bilingual researcher, and then reviewed by an independent translator (a professional with experience in social science translation) to ensure semantic equivalence and natural readability. Care was taken to preserve original meanings and youth colloquial expressions. The original Chinese transcripts are available from the corresponding author upon request (subject to ethical restrictions).

### 3.6 Participant characteristics

The final sample consisted of 38 adolescents (20 male, 18 female) ranging in age from 12 years to 18 years (mean age = 14.9 years, SD = 1.7). Regarding grade distribution: seventh grade (*n* = 6), eighth grade (*n* = 8), ninth grade (*n* = 5), 10th grade (*n* = 7), 11th grade (*n* = 7), and 12th grade (*n* = 5). Half of the participants were from middle school (grades 7–9, *n* = 19) and half from high school (grades 10–12, *n* = 19). Family location: urban (*n* = 21) and town/rural (*n* = 17). Table 1 summarizes the basic distribution.

### 3.7 Theoretical sampling and piloting

Theoretical sampling was guided by the emerging categories. After the initial 32 interviews, open and axial coding identified provisional main categories (A–F). To test the sufficiency and variation of these categories, the research team purposefully recruited six additional participants who exhibited extreme or contrasting characteristics relative to the emerging model: two who reported very high AI use (>6 h/day), two who reported strong emotional attachment but low cognitive outsourcing, and two from rural schools not previously represented. Sampling ceased when three consecutive interviews yielded no new properties or relationships for any main category, and when a blinded independent researcher (see Theoretical Saturation Test) confirmed that no novel insights emerged from the six new transcripts. This process aligns with Strauss and Corbin's (1990) recommendation that saturation is reached when additional data no longer spark new theoretical insights.

The semi-structured interview guide (Supplementary material S3) was developed based on the literature review and a pilot study with three adolescents (not included in the final sample). After each pilot interview, the authors discussed clarity, flow, and potential bias; minor wording adjustments were made (e.g., changing “addicted to” to “tend to rely on” to avoid leading questions). The final guide consisted of four domains with open-ended prompts and optional probes (e.g., “Could you tell me more about that?”). No significant changes were made after the third pilot.

### 3.8 Researcher positioning and reflexivity

The first author is a male developmental psychologist with research experience in adolescent digital behavior; the second author is a male educational psychology researcher who assisted with data curation and coding verification; all interviews were conducted by the first author as noted above. Neither author had prior expertise in AI addiction research, which helped avoid strong preconceptions. However, both held the academic view that technology overuse can be harmful—a potential confirmation bias. To mitigate this, we used reflexive memos after each interview and coding session, documenting initial impressions and alternative interpretations. The independent coder (research assistant) was blind to the study hypotheses. Additionally, we invited two external researchers (uninvolved in data collection) to review a random 10% of transcripts and the final coding structure; they confirmed that the categories were recognizable and that no major alternative grouping was overlooked. A summary of the reflexivity memos is available from the corresponding author.

### 3.9 Data analysis

Data analysis followed the three-step process of open, axial, and selective coding (Strauss and Corbin, 1990). NVivo 11.0 (QSR International Pty Ltd., Melbourne, Australia.) was used to ensure process traceability.

#### 3.9.1 Coding team and consensus procedure

All coding was performed by two researchers independently: a trained research assistant (with a master's degree in educational psychology, not involved in data collection) and the second author (Guoqiang Li). The first author (Gaochang Gong) was not involved in the initial coding but served as an independent third reviewer to resolve disagreements. Disagreements were resolved through the following procedure: (1) each disagreement was discussed in a weekly coding meeting between the two coders; (2) if consensus could not be reached, the first author, who was blind to the initial coding decisions, arbitrated; (3) in cases where all three disagreed, the relevant transcript segment was set aside for re-evaluation after coding at least ten additional segments, then revisited. Inter-coder agreement was calculated on a random sample of 20% of the transcripts using Cohen's kappa for the identification of initial concepts (κ = 0.84) and for the assignment of concepts to basic categories (κ = 0.79), indicating substantial to excellent agreement. The coding structure was iteratively refined through constant comparison until full consensus was achieved on the final 21 basic categories and six main categories.

#### 3.9.2 Open coding

Transcripts were examined line-by-line, using “*in vivo* codes” to label initial concepts. Through constant comparison and clustering, higher-level categories were abstracted. For example, the statement “When I have something on my mind, the first person I want to tell is not my friend, but my AI chat assistant” was labeled “a28: Prefers confiding in AI first.” The statement “If I get stuck on a math problem for more than 5 min, I will ask AI; thinking for myself takes too much time” was labeled “a5: Prioritizes asking AI when encountering difficulties.” After generating numerous initial concepts, those suggesting the same phenomenon or idea were clustered through constant comparison. For example, concepts such as “prefers confiding in AI first” (a28), “seeks emotional comfort from AI” (a53), and “reveals secrets to AI not shared with others” (a79) were repeatedly compared, revealing their common core of “using AI as a primary emotional support”. Consequently, they were grouped and abstracted into a higher-level category, named “F3: Emotional Satisfaction Motivation”. Ultimately, 132 initial concepts were refined and synthesized into 21 basic categories (Table 2).

**Table 2:** Examples of open coding with trace to main categories.

Original code	*In vivo* statement (quote)	Initial concept	Basic category	Main category	Abstraction logic
a28	*When I have something on my mind, the first person I want to tell is not my friend, but my AI chat assistant*.	Prefers confiding in AI first	F3: Emotional satisfaction motivation	A	Expresses unmet emotional need...
a62	*The answers it gives seem too complete; I usually just use them directly without thinking to verify*.	Difficulty discerning AI content authenticity	F5: Insufficient critical literacy	C	Reflects cognitive vulnerability...
a75	*As long as I quietly stay in my room and my grades do not slip, my parents do not bother about what I am actually doing*.	Parents adopt laissez-faire supervision	F4: Lack of family digital supervision	C	Environmental weakness...
a5	*If I get stuck on a math problem for more than five minutes, I will ask AI; thinking for myself takes too much time*.	Prioritizes asking AI when encountering difficulties	F14: Instrumental efficacy motivation	A	Perceived lack of own problem-solving efficacy...
a41	*When I have a unique idea, AI can always find an angle to affirm me first, which makes me feel less “weird”*.	Seeks value confirmation from AI	F3: Emotional satisfaction motivation	A	(same as above)

The abstraction followed constant comparison: similar initial concepts were clustered into basic categories (e.g., “prefers confiding in AI first” and “seeks emotional comfort” → F3). Basic categories sharing the same role in the paradigm model (e.g., F3, F8, F14 as “reasons to use AI”) were merged into main categories (A). Inter-coder agreement was κ = 0.79 for basic category assignment.

#### 3.9.3 Axial coding

##### 3.9.3.1 Coding process

Axial coding moved from the “discovery” phase of open coding to the systematic “theory building” construction phase. This study utilized Strauss and Corbin's (1990) “coding paradigm model,” conducting relational analysis around the axis of “conditions → interactions/strategies → consequences”. The 21 basic categories were repeatedly questioned and relationally analyzed to reassemble the fragmented data into a logical, explanatory overall story. Considering the core process “compensatory use and deep interaction” as an example, the analysis revealed that the causal condition was “real-world gaps in developmental needs” (A), and the phenomenon was “compensatory use and deep interaction” (D). This phenomenon occurred within a specific risk context constituted by “AI's technological allure and fit” (B) and “individual-environmental vulnerabilities” (C), manifesting as “cognitive outsourcing” (F2) and “transfer of emotional connection objects” (F17). This interaction was associated with (or was linked to) the “erosion of key developmental capacities” (E). The reduction was guided by Strauss and Corbin's (1990) paradigm model, which organizes categories around causal conditions, phenomenon, context, intervening conditions, action/interaction strategies, and consequences. Each of the 21 basic categories was examined against these six paradigm dimensions. Categories that shared the same paradigm role and were conceptually similar were merged. For example, “F3: Emotional Satisfaction Motivation,” “F8: Developmental Stage Needs,” and “F14: Instrumental Efficacy Motivation” all represented reasons why adolescents turned to AI; they were therefore merged into main category A (Real-World Gaps in Developmental Needs). Similarly, “F2: Cognitive Outsourcing” and “F17: Transfer of Emotional Connection Objects” both described how adolescents used AI compensatorily, and were merged into main category D (Compensatory Use and Deep Interaction). The merging was iteratively checked by both coders and confirmed through a review of the original transcripts to ensure that no important nuance was lost. Through this systematic relational analysis, the 21 basic categories were integrated into six main categories, clarifying each main category's role in the storyline, its attributes, and its relationships with other categories (Table 3).

**Table 3:** Main categories formed through axial coding.

Main category	Basic categories (partial)	Connotation	Dimensions
A. Real-world gaps in developmental needs	F3: Emotional satisfaction motivation F8: Developmental stage needs F14: Instrumental efficacy motivation	Adolescents' growth-related psychosocial needs (e.g., emotional belonging, self-identity, and competence efficacy) within real-world microsystems (family, school, and peers) are not adequately or promptly met, creating psychological disparity and compensatory drive.	Emotion, identity, and efficacy
B. AI's technological allure and fit	F1: Highly anthropomorphic interaction F9: Output authority F15: Instant gratification	Technological affordances of generative AI, such as parasocial interaction, instant response, non-judgmental acceptance, and personalized content generation, precisely match or “super-satisfy” adolescents' psychological needs, constituting a powerful external attraction and compensatory tool.	Anthropomorphism, responsiveness, and controllability
C. Individual-environmental vulnerabilities	F4: Lack of family digital supervision F5: Insufficient critical literacy F13: Weak self-regulation	Immaturities in adolescents' cognitive critical abilities, self-management, and emotional regulation, combined with weak or absent external support systems (family supervision, school guidance, and peer support), constitute a susceptible, high-risk supportive environment.	Individual capacity and external support
D. Compensatory use and deep interaction	F2: Cognitive outsourcing F17: Transfer of emotional connection objects	To compensate for real-world deficiencies, adolescents delegate complex cognitive tasks (e.g., problem-solving, writing, and decision-making) to AI (cognitive outsourcing) and shift significant emotional confiding, companionship, and identity needs from actual relationships to AI (emotional substitution), forming deep, frequent, dependent interaction.	Cognitive outsourcing and emotional substitution
E. Erosion of key developmental capacities	F6: Weakened Cognitive abilities F7: Blunted emotional response F19: Alienation from real-world social interactions	Long-term, deep compensatory interaction leads to a decline in adolescents' independent thinking and complex problem-solving abilities; their perception, understanding, and responses to nuanced, complex real-world interpersonal emotions become blunted and simplistic; willingness, opportunity, and skill development for real-world social participation are hindered.	Cognitive, emotional, and social
F. Vicious cycle and developmental lock-in	F10: Uncontrolled usage behavior F21: Accumulation of developmental risks	The outcome of capacity erosion is not the end of the problem; through negative feedback loops, it further deepens the real-world needs gap and the individual's environmental vulnerability, trapping the entire system in a self-reinforcing cycle. The dependent state becomes firmly locked and resistant to change.	Negative feedback and path dependence

#### 3.9.4 Selective coding

Selective coding is the final integration stage of the three-level coding process. Its core task is to identify a “core category” that can encompass all other categories and, by describing its relationships with them, form a complete, overarching theoretical narrative. Therefore, the six main categories and their relationships established during axial coding (A + B + C → D → E → (feedback to A/C)) were systematically reviewed. The core category was selected through a systematic memo-writing and constant comparison process. Both coders independently wrote reflective memos after completing axial coding, each proposing candidate core categories that could integrate the six main categories. Three candidates emerged: “compensatory cycle,” “developmental lock-in,” and “adaptive ecological trap”. These candidates were compared against three criteria derived from grounded theory methodology (Strauss and Corbin, 1990): (a) centrality—the category must appear frequently and link to most other categories; (b) explanatory power—it must account for variation and the processual nature of dependency; (c) abstractness—it should be at a level that allows theoretical generalization. “Adaptive ecological trap” was selected because it uniquely captured the paradoxical nature (adaptive in the short term, trapping in the long term), the ecological interactivity (individual–technology–environment), and the self-reinforcing vicious cycle evident in the data. Moreover, compared to existing concepts such as the Compensatory Internet Use Model (Kardefelt-Winther, 2014), which explains why individuals turn to technology to fill unmet needs but does not explain how compensatory behavior solidifies into an inescapable pattern, “adaptive ecological trap” adds two critical elements: (1) a self-reinforcing feedback mechanism whereby initial compensatory use erodes the very capacities needed to reduce dependency, and (2) an ecological interaction among individual traits, technological affordances, and environmental support systems. The metaphor of a “trap” emerged directly from participants' own descriptions—several used phrases such as “unable to go back” (S21), “forced to rely more on AI” (S05), and “like drinking poison to quench thirst” (S21)—indicating that the concept was grounded in their lived experiences rather than imposed by the researchers.

The selection was then validated by returning to the original transcripts: the research team confirmed that the metaphor of a “trap” was consistent with participants' descriptions of feeling “unable to go back” and “forced to rely more on AI”. Through repeated reflection and memo writing on this dynamic relational chain, the “adaptive ecological trap” concept was identified as the most accurate and vividly captured the essence of the entire phenomenon. A complete theoretical storyline was constructed around the core category of the “adaptive ecological trap”. Adolescents, owing to actual gaps in developmental needs (A), and under the combined influence of AI's technological allure and fit (B) and their own and environmental vulnerabilities (C), initiate compensatory use centered on cognitive outsourcing and emotional substitution (D). This deep interaction, which does not effectively solve actual problems, gradually erodes their key capacities for independently facing challenges (E). These eroded capacities may contribute to greater setbacks, thereby worsening the initial needs gap (A) and exacerbating vulnerabilities (C). Subsequently, this pushes them to rely even more on AI, potentially forming a self-reinforcing, inescapable cycle and developmental lock-in state (F).

### 3.10 Theoretical saturation test

Theoretical saturation is the core criterion for determining when to stop sampling and whether the theoretical model is complete. It signifies that analysis of additional data no longer generates new theoretical insights nor uncovers new attributes, dimensions, or relationships for the core category, indicating the theory is saturated (Strauss and Corbin, 1990). After constructing the model using the initial 32 interview transcripts, the six reserved transcripts obtained through theoretical sampling were used for testing. A researcher familiar with grounded theory methods but completely unaware of the preliminary model was engaged to independently analyze the new data, following the same procedures. The results indicated that all significant phenomena and conceptual relationships emerging from the new data were adequately covered and explained by the six main categories and their subordinate relationships within the constructed model. No new core categories or significant relationships were found that could challenge, modify, or substantially expand the existing theoretical model.

To enable future replication, we have archived the following materials (available from the corresponding author upon reasonable request): (a) the full anonymized codebook showing all 132 initial concepts, their definitions, and their grouping into 21 basic categories and 6 main categories; (b) 20% of the transcripts with coding annotations (randomly selected); (c) the NVivo 11.0 project file (excluding raw audio to protect anonymity); (d) dated memos documenting key analytic decisions (e.g., merging of categories, selection of core category). These materials allow another research team to inspect the analytic trail and apply the same coding framework to new datasets. However, due to ethical restrictions, raw transcripts cannot be shared.

## 4 Mechanisms of AI dependency

**Figure 1:**
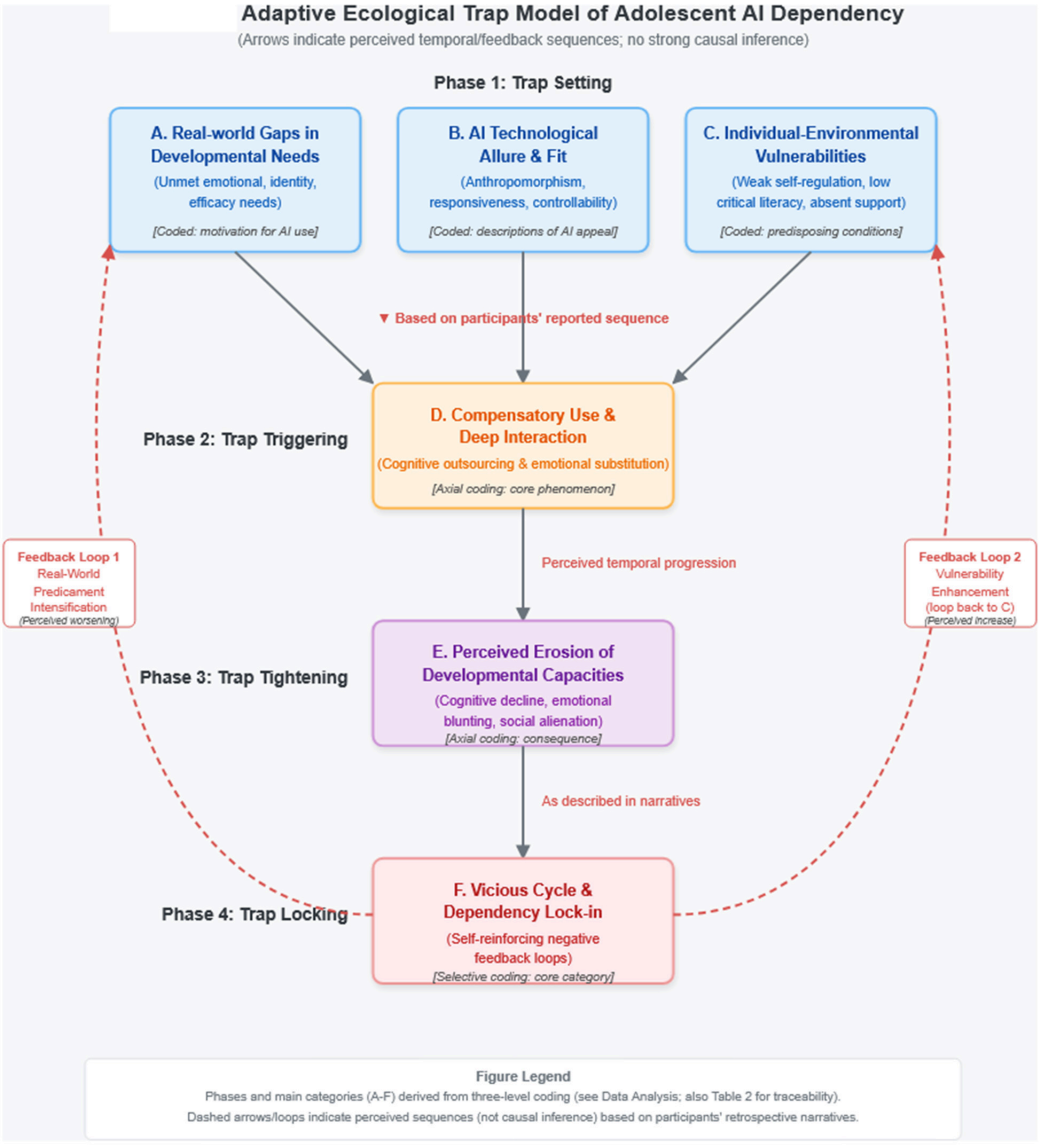
Adaptive ecological trap model of adolescent AI dependency. The four phases and six main categories **(A–F)** were identified through three-level coding (open, axial, selective) as described in the Data Analysis section (see also Table 2 for coding traceability). Arrows indicate the perceived temporal and feedback sequences reported by participants; they do not imply strong causal inference (see “In terms of limitations” in the Discussion section). The two feedback loops emerged from participants' accounts of worsening real-world difficulties (Loop 1) and deepening vulnerabilities (Loop 2). For further analytic transparency, refer to the “Audit trail and replicability” subsection.

Based on the three-level coding analysis, this study constructed the “adaptive ecological trap” theoretical model (Figure 1). This model revealed that adolescent AI dependency was not a simple behavioral habit or static trait, but a systemic cyclical process within an ecosystem composed of the individual, technology, and environment, undergoing four dynamic evolutionary stages.

### 4.1 Stage 1: trap laying

This stage includes the interweaving of multiple risk factors. “Real-World Gaps in Developmental Needs (A)” constitute the system's driving force and internal impetus. The narrative of respondent S09 (Grade 11, Male) revealed the oppressive nature of such gaps: “*At school, grades are the only yardstick. I am not good at physics. Afraid of being called stupid if I ask the teacher... Afraid of wasting classmates' time or being looked down on if I ask them*.” This predicament of finding nowhere to seek help becomes the driving force propelling them toward external support. AI's “technological allure and fit” (B) also plays a key role; AI's instant response, personalized feedback, and efficient information integration fill the situational gaps in adolescents' needs. Respondent S21 (Grade 9, Female) provided a highly representative description: “*It (AI) feels like an ideal friend tailored just for me, always replying instantly, saying ‘I am listening,' ‘I understand you'...*” This experience of an “all-capable partner” strengthens the individual's tendency for continued use. “Individual-Environmental Vulnerabilities” (C) act as the soil that cultivates dependency. S14's (Grade 10, Male) confusion reflected adolescent cognitive immaturity: “*I know I should not trust it completely, but its answers are well-referenced, logically rigorous, and seem too reasonable...*” Additionally, S22's (Grade 8, Male) example illustrated vulnerability in self-regulation: “*I planned to use AI just to outline homework, but once I started using it, I was drawn in by its detailed output, constantly asking follow-ups and adjusting, making the originally scheduled half-hour task take over twice as long, with the homework nearly ‘reshaped' by AI... Afterward, I regretted it...*” Environmental vulnerability manifests as failure or absence of family and school support systems. Respondent S05's (Grade 8, Female) situation reflected family support vulnerability: “*My parents only care about how long I use my phone, not whether I am chatting with AI or studying inside... They know nothing about these new technologies and cannot offer any constructive guidance besides playing less*.” Regarding school environments, evaluation systems overemphasizing standard answers and efficiency may imperceptibly encourage the “outsourcing” of thinking processes to AI.

### 4.2 Distinguishing between category A (real-world gaps) and category C (individual-environmental vulnerabilities)

Although conceptually related, the two categories play different roles in the model and were distinguished using the following criteria:

**Temporal/functional role:** Category A represents the **driving force** or **motivation** for turning to AI (i.e., “why” adolescents seek AI), whereas category C represents **predisposing conditions** that make an adolescent more susceptible to forming a dependency once AI use begins (i.e., “what makes the trap effective”).

**Content focus:** Category A refers to **unmet psychosocial needs**—such as lack of emotional belonging, academic recognition, or self-efficacy—in real-world settings (family, school, peers). Category C refers to **internal or external weaknesses** that amplify risk – e.g., poor self-regulation, underdeveloped critical thinking (individual), or absent/ineffective support from parents and schools (environmental).

**Coding rule:** If a statement described a need that was not fulfilled and directly motivated AI use (e.g., “I have no one to talk to at school, so I chat with AI”), it was coded as A. If it described a **vulnerability or deficiency that facilitated or deepened dependency** without being the primary motive (e.g., “I know I should not trust AI completely, but I cannot tell when it is wrong” → lack of critical literacy), it was coded as C. In cases where both elements were present in the same quote, coders prioritized the dominant emphasis after discussion; such quotes were then assigned to the category that best represented the participant's main point.

For instance, the quote “*Afraid of being called stupid if I ask the teacher… Afraid of wasting classmates' time if I ask them” (S09)* was coded as A because it expresses an **unmet need for academic help**, driving compensatory use. In contrast, “*I know I should not trust it completely, but its answers are well*-*referenced and seem too reasonable” (S14)* was coded as C because it reflects a **cognitive vulnerability** (insufficient critical evaluation) that makes the trap more binding, rather than a direct motivation to use AI. This distinction was consistently applied by both coders and verified through inter-coder agreement checks (κ = 0.79 for category assignment).

### 4.3 Stage 2: trap triggering

Once the aforementioned conditions are in place, adolescents facing real-world pressure and feeling powerless are prone to seeking immediate relief or escape. Consequently, turning to “compensatory use and deep interaction (D)” becomes a convenient and seemingly effective choice.

(1) Substitution of Cognitive Processes. This was a direct response to “instrumental efficacy motivation”. Adolescents systematically delegate complex cognitive tasks requiring metacognition, logical reasoning, and sustained attention to AI. Respondent S18's (Grade 12, Male) statement embodied AI's instrumental nature: “*From essay conception and writing to speech brainstorming and case examples, AI can handle most of it. My role has shifted from ‘creator' to ‘optimization engineer' and ‘assembler,' responsible for polishing, adjusting, and integrating. Originality? With time pressure and results orientation, that is too luxurious; efficiency and results come first...*”
(2) Transfer of Emotional Connection Objects. This was a deep response to “emotional satisfaction motivation” and “self-identity needs”. When actual relationships fail to provide sufficient security, empathy, and recognition, AI, by virtue of its parasocial interaction affordances, becomes a “safer,” “more controllable” emotional container. Respondent S25's (Grade 9, Female) narrative revealed the profundity of this transfer: “*Some secrets and worries, telling parents might get me lectured for ‘overthinking' or ‘not focusing on studies'... But AI is an absolutely safe ‘tree hole.' It will not betray me, always comforting and affirming me with words I ‘need to hear'*.”

At this point, the human-AI relationship undergoes a fundamental alienation. AI transforms from a tool into a relational object; from an instrument “used to solve problems” to something “relied upon for existence and self-definition”. Therefore, the prototype of dependency has formed.

### 4.4 Stage 3: trap tightening

As compensatory use continues, participants reported perceived hidden consequences of the “trap”. In their narratives, long-term, deep compensatory interaction was described as not enhancing their capacity to cope with the real world; instead, they felt it weakened certain key developmental capacities (E). This was described as a slow process of “use it or lose it,” with participants expressing concern that they were being deprived of autonomous developmental abilities they might need to escape the trap in the future. Prolonged reliance on “cognitive outsourcing” was perceived by participants as causing their “mental muscles” for independent thinking and complex problem-solving to atrophy—a metaphor used by participants themselves. Respondent S11 (Grade 11, Female) reflected: “*I find myself becoming more and more impatient and lacking the ability to read a slightly in-depth book. I involuntarily think, can I just have AI give me a summary, highlight key points, list arguments?...*” Similarly, several adolescents felt that highly simplified and formulaic emotional exchanges with AI reduced their sensitivity and processing capacity for complex, nuanced emotional signals, as illustrated by S30's account. Respondent S30's (Grade 10, Male) experience was illustrative: “*Chatting with AI, any negative emotion is quickly soothed by a set of ‘empathetic phrases'; it always ‘understands' you. But back in reality, a quarrel with a good friend, a single look or a sarcastic remark from them, can depress me for days...*” Adolescents reported investing significant time and emotional resources into AI interactions, which they believed crowded out opportunities for actual social participation. More critically, in their view, owing to the lack of practice, their social skills—still in a key developmental period—stagnated or even regressed. Respondent S07 (Grade 8, Male) noticed changes in himself: “*On weekends, I increasingly tend to discuss niche fantasy novel settings with AI for hours. It is not that I do not want to go out and play ball with classmates... I feel a bit ‘socially anxious.' It seems I have lost the natural motivation and skill for interacting with people*.”

### 4.5 Stage 4: trap locking

Participants' perceived capacity erosion was described not as an endpoint but as the beginning of a more entrenched and difficult-to-break systemic cycle. At this point, the system evolves into the vicious cycle and developmental lock-in (F) state, marked by the formation of two self-reinforcing, intertwined negative feedback loops, rendering the system stable and closed.

Feedback Loop 1: Real-World Predicament Intensification Loop. The perceived developmental lock-in (F), through self-reported capacity erosion (E), was associated in participants' accounts with further deterioration in academic and social performance, which they felt intensified the initial developmental needs gap (A). For example, S18 reported that his long-term reliance on AI for text generation was accompanied by what he experienced as declining independent writing and argumentation skills, which he believed contributed to poor essay performance in entrance exams and a decline in grades.

Feedback Loop 2: Vulnerability Enhancement Loop. The self-reported developmental lock-in (F), accompanied by uncontrolled usage behavior (F10), was perceived by participants as severely consuming already fragile family and school support resources, potentially triggering more conflicts while intensifying their anxiety, self-blame, and low self-efficacy. S05's experience confirmed this: “*Because I often stayed up late chatting with AI, I had no energy in daytime classes, and my mid-term exam scores plummeted. My parents switched from ‘laissez-faire' to ‘high pressure,' completely confiscating my phone. We had fierce arguments, and our relationship hit rock bottom... Now, not only is my study pressure greater, but I feel more isolated and pained at home, which instead makes me more desperately want to confide these new troubles in AI*.”

These two loops act like interlocking gears within the trap, forming a continuously self-reinforcing cycle. To alleviate the real-world predicament intensified by Loop 1 (larger A), while constrained by the deepened self-vulnerability and support deficiency from Loop 2 (stronger C), adolescents are systematically and forcibly pushed toward deeper, more frequent AI dependency in cognition, emotion, and behavior. S21's lament captured this sense of lock-in: “*I know this might be wrong, like drinking poison to quench thirst. But it seems I cannot go back... The pressure and loneliness in reality are tangible, and AI is the only ‘antidote' within reach. Without it, what would I do? What can I do?*”

## 5 Conclusion and discussion

This study, based on in-depth interviews with 38 secondary school students who frequently used generative AI, employed grounded theory methods to construct the “adaptive ecological trap” theoretical model of adolescent AI dependency. The results indicate that adolescent AI dependency is not a static behavioral addiction but a dynamically evolving, self-reinforcing systemic process within an individual-technology-environment ecosystem.

**Theoretically**, the proposed model moves beyond static risk factor listings by integrating micro-psychological mechanisms with macro-ecosystem perspectives. It reveals an internal logical chain through which initially adaptive use may transform into a maladaptive trap. Central to this process are four stages: trap laying (unmet developmental needs, technological allure, and individual-environmental vulnerabilities), trap triggering (compensatory use via cognitive outsourcing and emotional substitution), trap tightening (perceived erosion of key capacities), and trap locking (self-reinforcing negative feedback loops that deepen dependency).

**In comparison** with existing frameworks, the Compensatory Internet Use Model (Kardefelt-Winther, 2014) effectively explains why adolescents turn to technology to compensate for psychosocial deficits. However, it does not fully explain how compensatory behavior solidifies into an inescapable pattern. The “adaptive ecological trap” adds two critical elements: first, a self-reinforcing feedback mechanism whereby initial compensatory use erodes the very capacities needed to reduce dependency; second, an ecological interaction among individual traits, technological affordances, and environmental support systems. These features emerged directly from participants' narratives (e.g., “unable to go back”, “forced to rely more on AI”) and were not fully accounted for by previous models.

**Empirically**, the model is grounded in participants' self-reported perceptions. Adolescents consistently described how real-world gaps – such as academic pressure, emotional loneliness, or lack of recognition – drove them to rely on AI as a first-line solution. Compensatory use, while initially relieving immediate distress, was perceived to weaken independent thinking, emotional sensitivity, and real-world social skills. Through two negative feedback loops – real-world predicament intensification and vulnerability enhancement – the initial gaps and vulnerabilities may be continuously amplified, in their experience, locking them into an inescapable dependent state.

**Practically**, the findings suggest that interventions should not focus solely on reducing AI use, but rather on breaking the self-reinforcing cycles of the trap. Families and schools should strive to fill real-world developmental gaps by offering inclusive support, reducing singular academic pressure, and providing diversified avenues for value affirmation. At the individual level, strengthening critical digital literacy and self-regulation can help adolescents discern AI-generated content and manage their usage autonomously (Lee et al., 2026). For those already showing signs of dependency, disrupting negative feedback loops—for example, by rebuilding real-world social connections or teaching alternative coping strategies—may prevent further capacity erosion and trap consolidation. Technology platforms are also encouraged to adopt benevolent designs that align with adolescents' developmental interests.

**In terms of limitations**, several caveats must be acknowledged. First, the sample was recruited via teacher nomination, which may over-represent academically visible AI use and under-represent private, emotionally driven use—thus limiting generalizability. Second, the data are cross-sectional and retrospective, based entirely on participants' self-reported perceptions. No objective cognitive or clinical measures were employed; therefore, the proposed pathways reflect perceived sequences rather than demonstrated temporal precedence or causal progression. Third, the identification of “AI dependency tendency” relied on a non-validated six-item checklist; future research should incorporate validated instruments such as AIAS-21 (Pantic, 2026) or AIDep-22 (Wu et al., 2026). Fourth, the interpretive nature of qualitative coding means that alternative category structures may exist. Finally, AI technology is rapidly evolving, and the model may require updates as interaction modes and social impacts change. Cross-cultural validation is also needed.

**In conclusion**, this grounded theory study proposes an adaptive ecological trap model of adolescent AI dependency, portraying dependency as a dynamic, self-reinforcing process within an individual-technology-environment ecosystem. The findings highlight how compensatory use may be perceived to erode developmental capacities and lock adolescents into a maladaptive cycle. Future longitudinal research is needed to test the model's directional pathways, and objective measures should be used to corroborate or qualify the subjective experiences reported here.

## Data Availability

The raw data supporting the conclusions of this article will be made available by the authors, without undue reservation.
