# Supplementary material for: The adaptive ecological trap: a grounded theory study of adolescent AI dependency
Source: Front Psychol. 2026 Jun 18;17:1839672. doi: 10.3389/fpsyg.2026.1839672 (PMC13323298; doi:10.3389/fpsyg.2026.1839672)
Supplement: Supplementary file 1 [file Table_1.docx]

**Teacher Behavioral Anchor Guide for Nominating Students with Possible AI Dependency Tendency**

*(For internal use only – not a diagnostic tool)*

Dear Teacher,

Please read the following observable indicators. If a student has shown **at least two** of these behaviors consistently over the past two months, please consider nominating them for the study. There is no need to make a clinical judgment; simply check the indicators you have observed.

**Indicators (check all that apply):**

□ **Priority use** – The student often uses generative AI tools (e.g., ChatGPT, DeepSeek, Doubao) to complete homework or assignments that peers of similar ability finish independently without AI assistance.

□ **Emotional reliance** – The student appears visibly anxious, frustrated, or upset when AI access is limited (e.g., during no‑phone rules in class or at home).

□ **Social withdrawal** – The student prefers interacting with AI chatbots over face‑to‑face conversations with classmates during breaks or after school.

□ **Neglect of other activities** – The student’s participation in sports, hobbies, or family activities has decreased noticeably, and they attribute this decline to spending time on AI.

□ **Self‑regulatory difficulty** – The student reports (or is observed) intending to use AI for a short time but repeatedly spends much longer, expressing regret afterward.

□ **Academic decline** – There is a noticeable drop in the quality of original writing or problem‑solving that the teacher suspects is linked to overreliance on AI‑generated answers, although grades alone are not sufficient evidence.

**Student name (optional for nomination form): _________________________**

**Indicators observed (list numbers): _________________________**

**Teacher signature: ______________________ Date: ___________________**
