# Supplementary material for: The adaptive ecological trap: a grounded theory study of adolescent AI dependency
Source: Front Psychol. 2026 Jun 18;17:1839672. doi: 10.3389/fpsyg.2026.1839672 (PMC13323298; doi:10.3389/fpsyg.2026.1839672)
Supplement: Supplementary file 2 [file Table_2.docx]

**Student Screening Checklist for AI Dependency Tendency**

(*To be administered orally by the researcher*)

**Instructions:** Please answer each question with “Yes” or “No” based on your experience with generative AI (e.g., ChatGPT, DeepSeek, Doubao, etc.) in the past three months. There are no right or wrong answers.

| # | Item | Yes | No |
| --- | --- | --- | --- |
| 1 | When facing a difficult problem (study or personal), do you usually go to AI first before trying to solve it yourself? | □ | □ |
| 2 | Do you use generative AI almost every day and feel uncomfortable if you miss one day? | □ | □ |
| 3 | Do you feel upset, restless, or bored if you cannot use generative AI for a day? | □ | □ |
| 4 | Has your use of AI ever caused your grades to drop or made you spend less time with friends/family? | □ | □ |
| 5 | Do you find it difficult to stop using AI even when you know you have other important things to do? | □ | □ |
| 6 | Have you ever lied to your parents or teachers about how much you use AI? | □ | □ |

**Scoring:** Count the number of “Yes” responses. A total score of 4 or higher indicates a clear tendency towards AI dependency for the purpose of this study.

**Researcher’s note:** This checklist is a study‑specific screening tool and has not been clinically validated. Its purpose is to select participants for qualitative interviews, not to diagnose a disorder.
