# Supplementary material for: The adaptive ecological trap: a grounded theory study of adolescent AI dependency
Source: Front Psychol. 2026 Jun 18;17:1839672. doi: 10.3389/fpsyg.2026.1839672 (PMC13323298; doi:10.3389/fpsyg.2026.1839672)
Supplement: Supplementary file 3 [file Table_3.docx]

**Semi‑structured Interview Guide (English translation)**

**Introduction to participant:**

“*Thank you for agreeing to talk with me. This interview is about how secondary school students use generative AI (like DeepSeek, Doubao, ChatGPT, etc.). There are no right or wrong answers. Everything you say will be kept confidential. You can skip any question or stop at any time.”*

**Domain 1: AI usage history and patterns**

1. How long have you been using generative AI? Which tools do you use most often?

2. On a typical day, how much time do you spend using AI? For what purposes (homework, chat, entertainment, information search, etc.)?

3. Can you describe a typical situation when you open an AI app?

**Domain 2: Motivations and perceived benefits**

1. Why do you use AI rather than asking a teacher, a classmate, or searching by yourself?

2. What do you like most about AI? What makes you keep using it?

3. Can you give me an example of a time when AI helped you solve a difficult problem or made you feel better emotionally?

**Domain 3: Emotional and cognitive experiences with AI**

1. How do you feel when you are interacting with AI? Do you feel that AI understands you? In what way?

2. Have you ever shared something with AI that you would not share with your parents or friends? Can you tell me about it (without revealing specifics if you prefer)?

3. Do you feel that your thinking or problem‑solving habits have changed since you started using AI frequently?

**Domain 4: Negative consequences and reflections**

1. Have you ever felt that AI use caused problems for you – for example, in your grades, relationships with family or friends, sleep, or how you feel about yourself?

2. Have you tried to reduce your AI use? What happened? Was it easy or difficult?

3. Looking back, do you think your AI use is more helpful or more harmful? Why?

**Closing prompt:**

*“Is there anything else you would like to add about your experience with AI that we haven’t talked about?”*

*Note: Probes such as “Could you tell me more about that?” or “What happened next?” were used flexibly to encourage elaboration.*
